# Supplementary material for: A Computational Model for Histone Mark Propagation Reproduces the Distribution of Heterochromatin in Different Human Cell Types
Source: PLoS One. 2013 Sep 19;8(9):e73818. doi: 10.1371/journal.pone.0073818 (PMC3777982; doi:10.1371/journal.pone.0073818)
Supplement: File S1 — File includes Figures S1–S16. Figure S1. Distribution of histone modifications measured with ChIP-seq experiments on human CD4 T cells for chromosome 2. Figure S2. Distribution of histone modifications measured with ChIP-seq experiments on human CD4 T cells for chromosome 3. Figure S3. Distribution of histone modifications measured with ChIP-seq experiments on human CD4 T cells for chromosome 17. Figure S4. Distribution of histone modifications measured with ChIP-seq experiments on human HeLa cells for chromosome 1. Figure S5. Distribution of histone modifications measured with ChIP-seq experiments on human HeLa cells for chromosome 2. Figure S6. Distribution of histone modifications measured with ChIP-seq experiments on human HeLa cells for chromosome 3. Figure S7. Distribution of histone modifications measured with ChIP-seq experiments on human HeLa cells for chromosome 17. Figure S8. Pearson's correlation between simulations and experiments on chromosome 1 on HeLa cells for different values of the parameters , and . No data was available for the marks H3K18ac and H3K23ac (fields remain black). The results are almost identical to the ones for CD4+ cells. Figure S9. Pearson's correlation between simulations and experiments on chromosomes 1–3 ad 17 on CD4+ cells for different values of the parameters , and . The results are very similar between the different chromosomes. Figure S10. Pearson's correlation between simulations and experiments on chromosome 1 on CD4+ T cells for different values of the parameters , and . We used wrong coordinates for the nucleation sites based on human genome hg19 instead of hg18. Resulting correlation values are much lower than for correct nucleation site positions. Hence, these slightly differently positioned sites impede accurate reproduction of the chromatin domains for all parameter values. Figure S11. Comparing the simulation results for chromosomes 2,3 and 17 to the CD4+ T cells data set. Parameters are the same as in Fig. 5. Fig [file pone.0073818.s001.pdf]

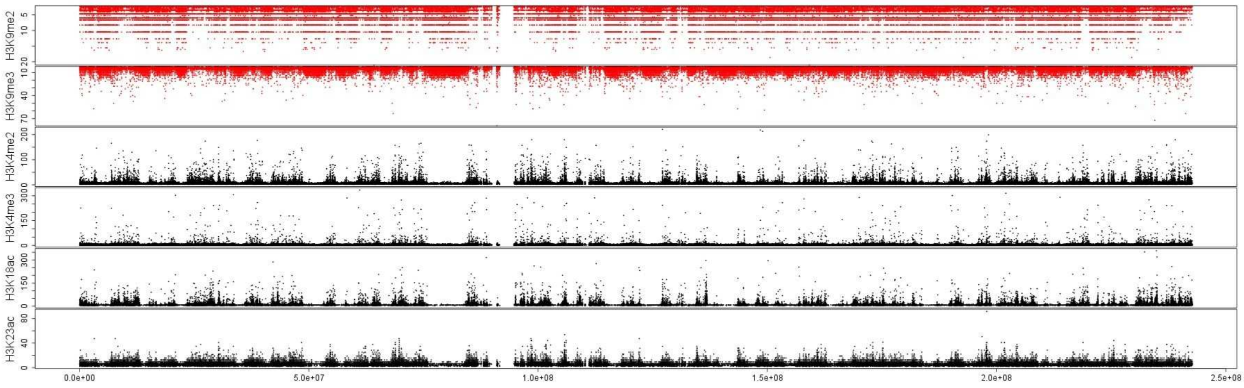

Figure S1. Distribution of histone modifications measured with ChIP-seq experiments on human CD4+ T cells for chromosome 2.

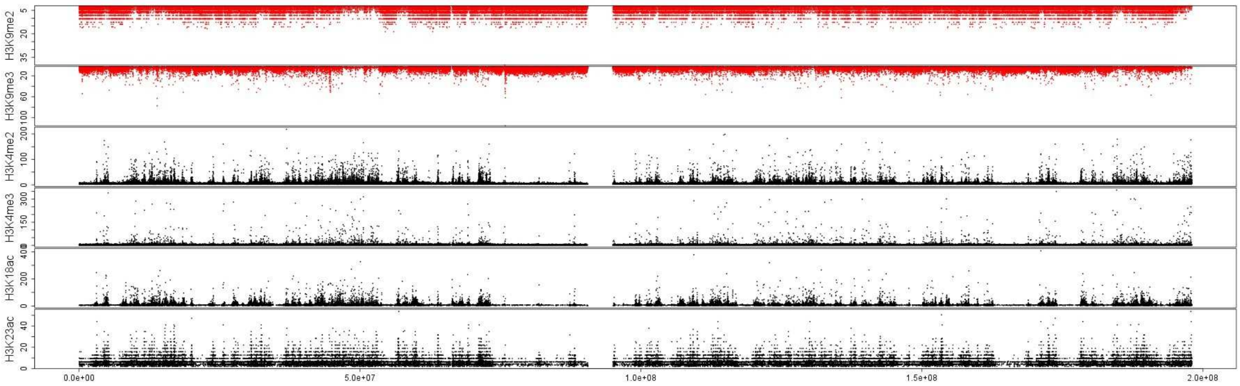

Figure S2. Distribution of histone modifications measured with ChIP-seq experiments on human CD4+ T cells for chromosome 3.

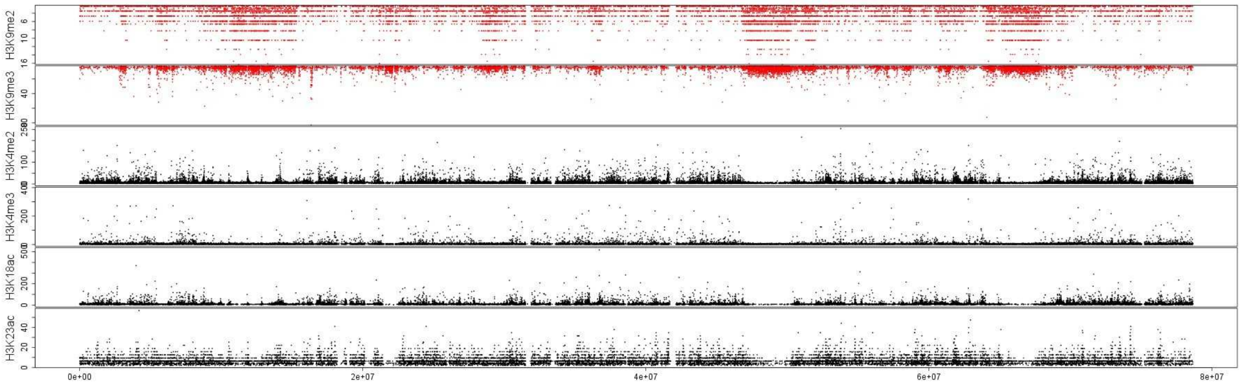

Figure S3. Distribution of histone modifications measured with ChIP-seq experiments on human CD4+ T cells for chromosome 17.

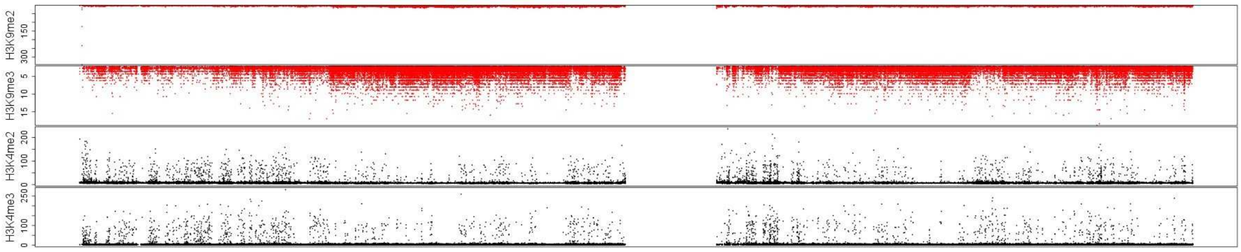

Figure S4. Distribution of histone modifications measured with ChIP-seq experiments on human HeLa cells for chromosome 1.

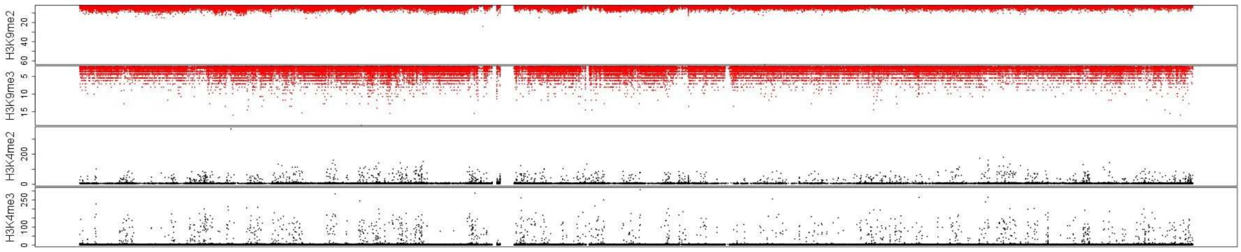

Figure S5. Distribution of histone modifications measured with ChIP-seq experiments on human HeLa cells for chromosome 2.

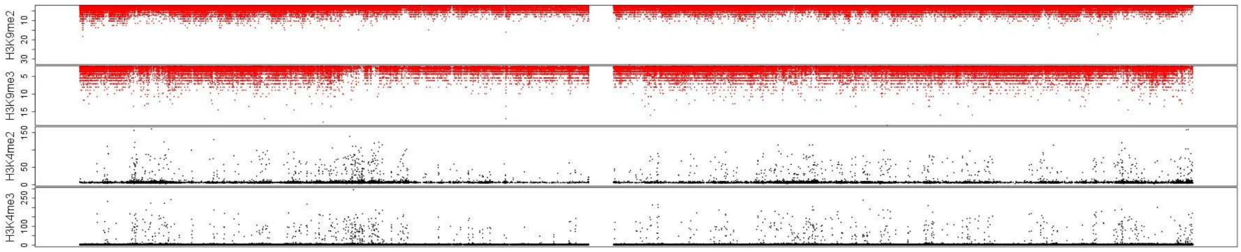

Figure S6. Distribution of histone modifications measured with ChIP-seq experiments on human HeLa cells for chromosome 3.

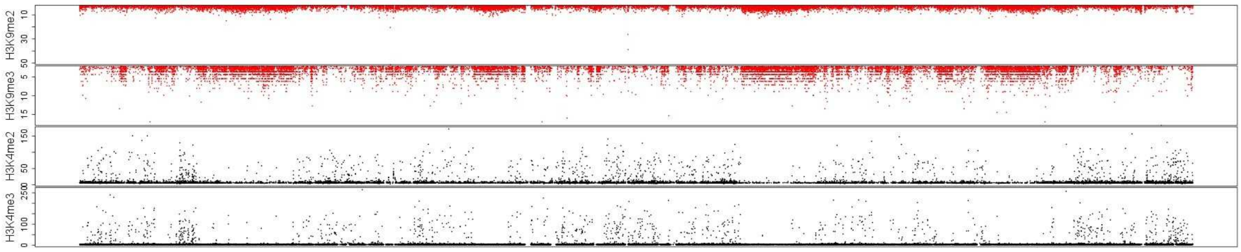

Figure S7. Distribution of histone modifications measured with ChIP-seq experiments on human HeLa cells for chromosome 17.

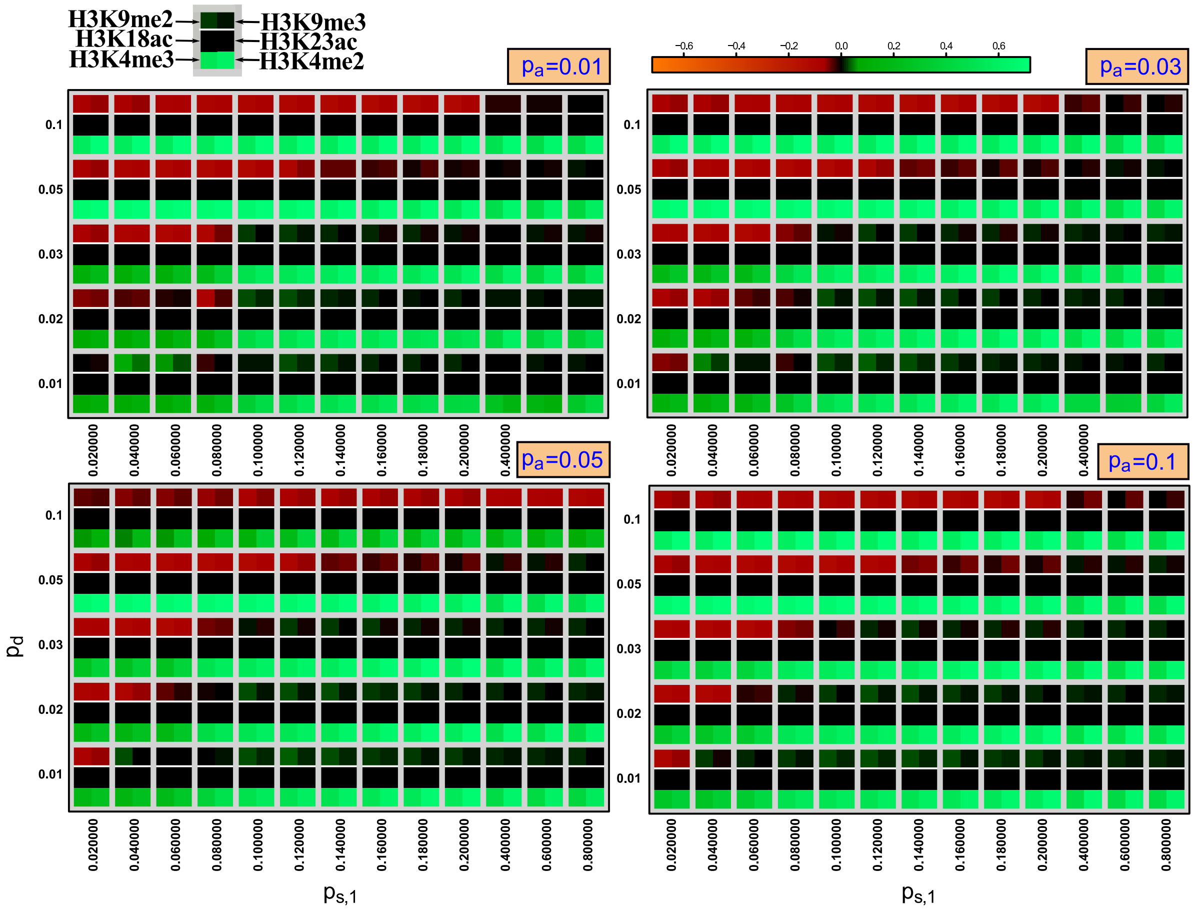

Figure S8. Pearson's correlation between simulations and experiments on chromosome 1 on HeLa cells for different values of the parameters $p_{s,1}$, p_d and p_a. No data was available for the marks H3K18ac and H3K23ac (fields remain black). The results are almost identical to the ones for CD4+ cells.

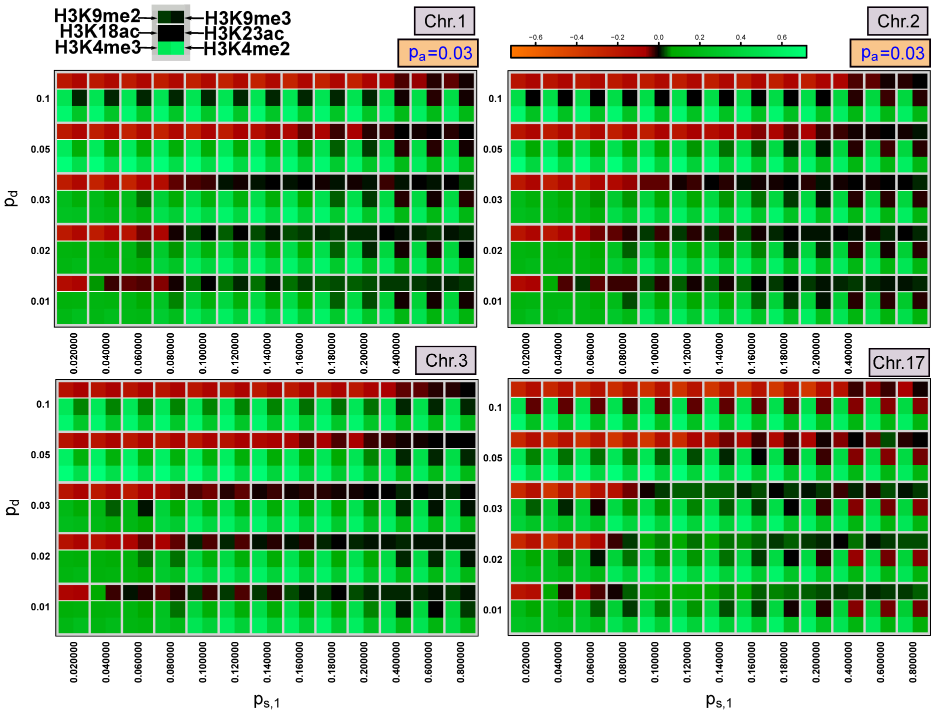

Figure S9. Pearson's correlation between simulations and experiments on chromosomes 1-3 ad 17 on CD4+ cells for different values of the parameters $p_{s,1}$, p_d and $p_a = 0.03$. The results are very similar between the different chromosomes.

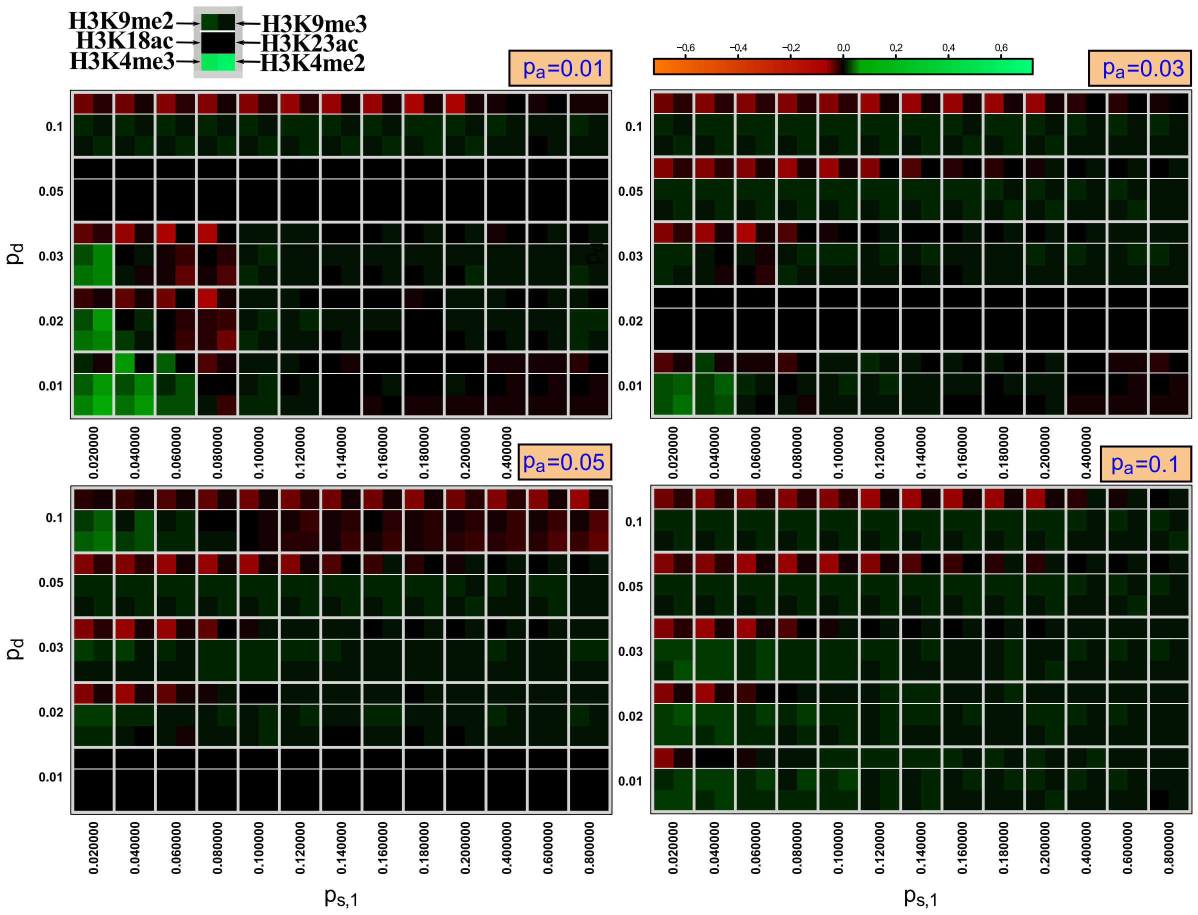

Figure S10. Pearson's correlation between simulations and experiments on chromosome 1 on CD4+ T cells for different values of the parameters $p_{s,1}$, p_d and p_a. We used wrong coordinates for the nucleation sites based on human genome hg19 instead of hg18. Resulting correlation values are much lower than for correct nucleation site positions. Hence, these slightly differently positioned sites impede accurate reproduction of the chromatin domains for all parameter values.

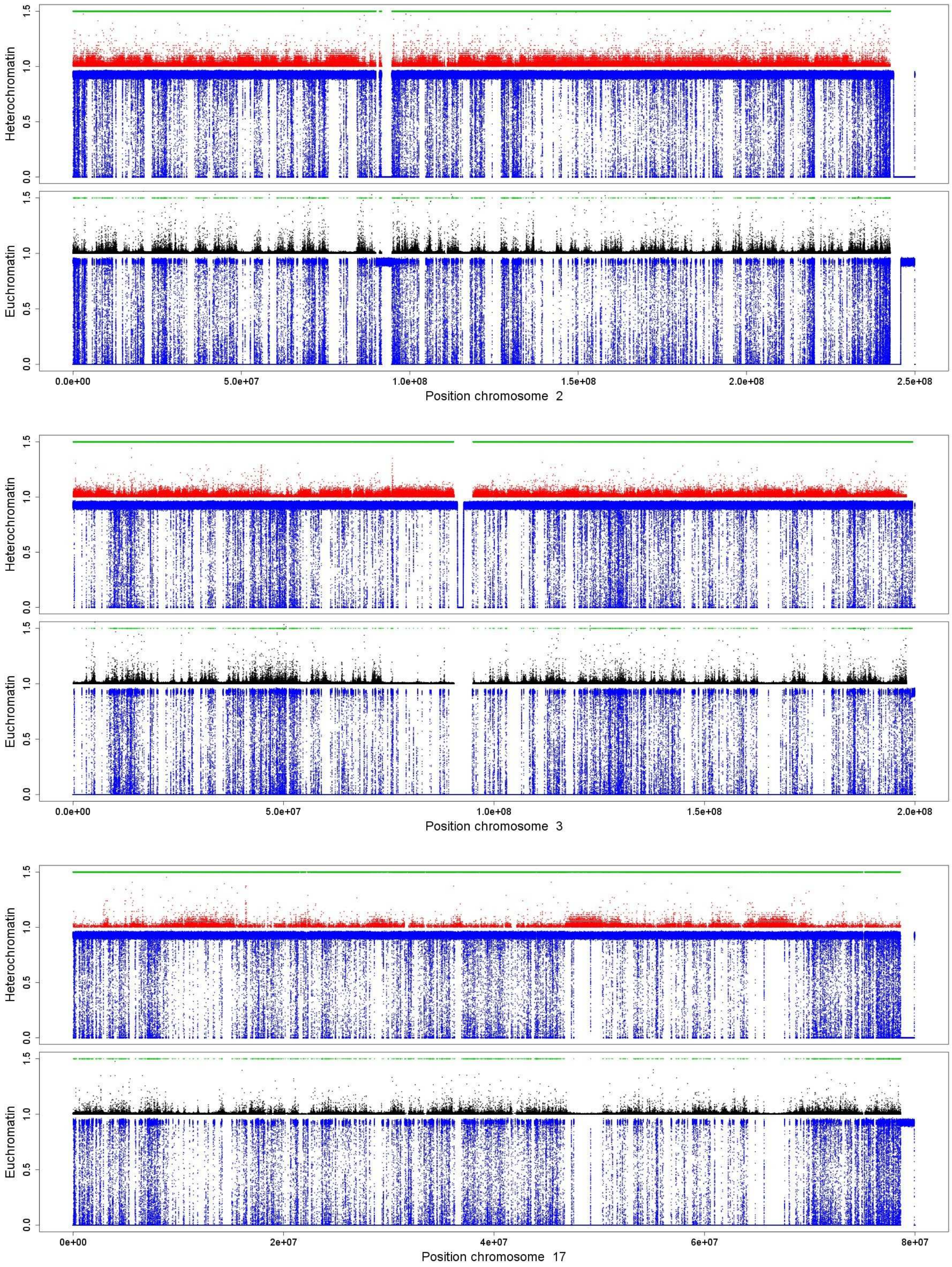

Figure S11. Comparing the simulation results for chromosomes 2,3 and 17 to the CD4+ T cells data set. Parameters are the same as in Fig. 5.

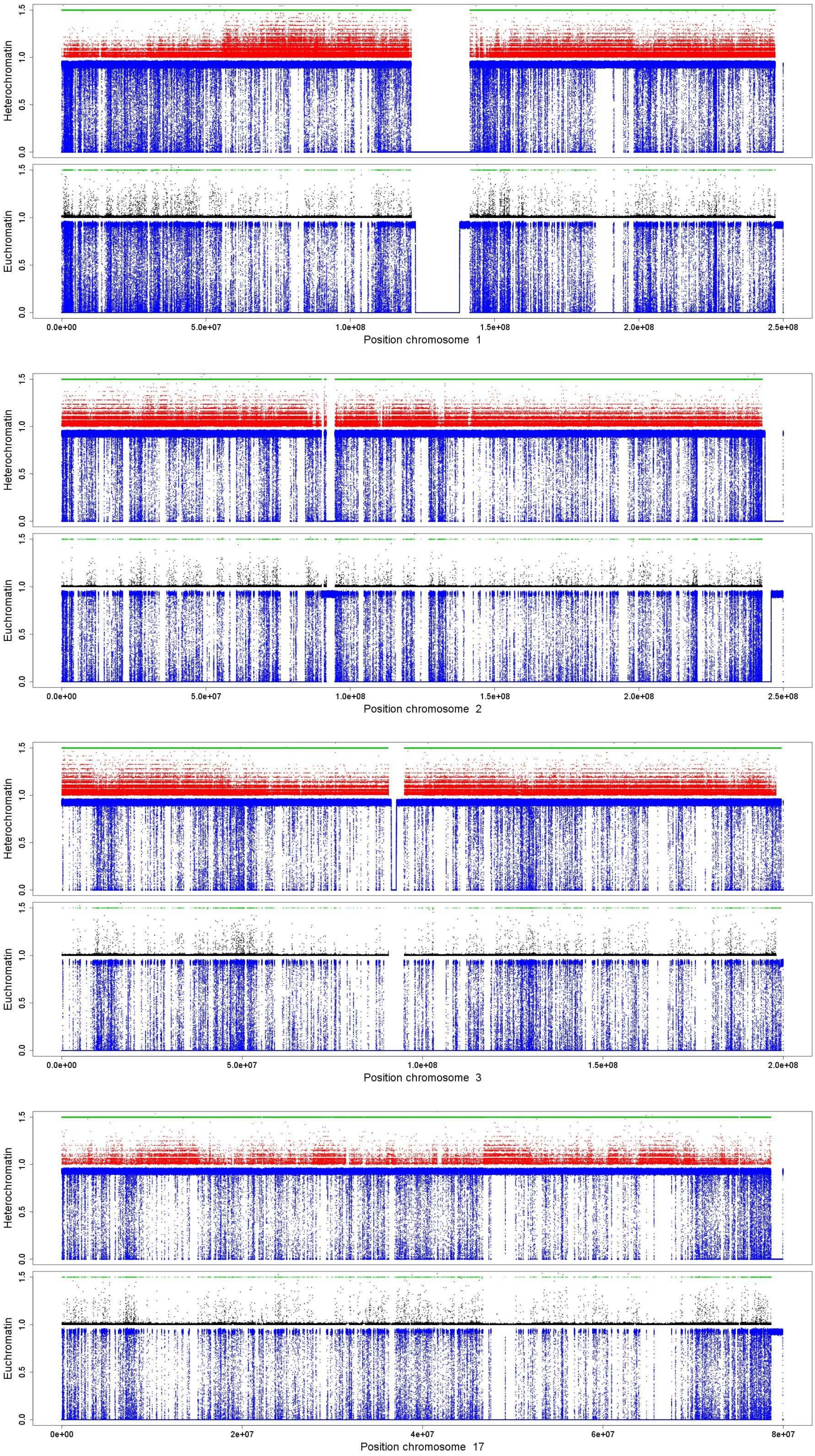

Figure S12. Comparing the simulation results for chromosomes 1-3 and 17 to the HeLa cells data set. Parameters are the same as in Fig. 5.

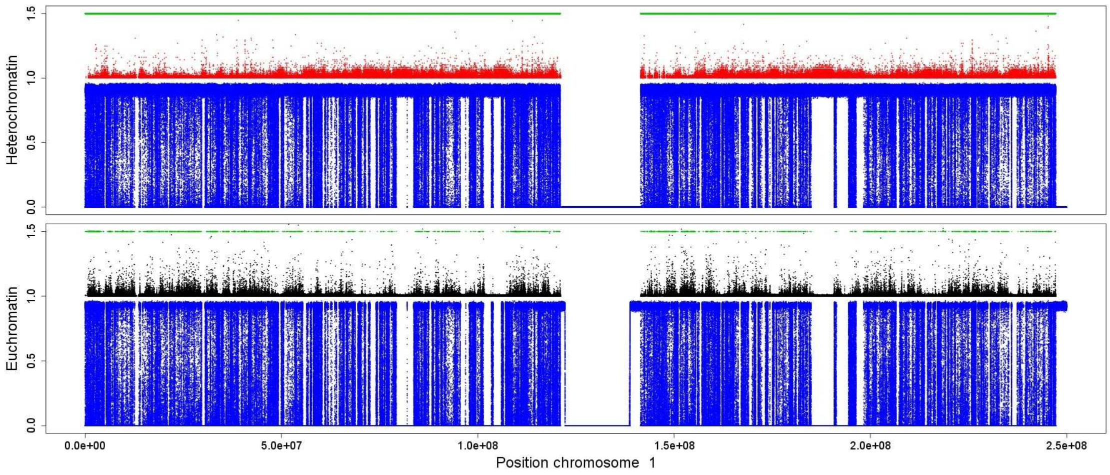

Figure S13. Comparing the simulation results for chromosome 3 to the CD4+ ChIP-seq data set. Parameters are the same as in Fig. 5 except of a smaller propagation rate for heterochromatin, $p_{s,1} = 0.08$ leading to an aberrant state of chromatin domain distribution.

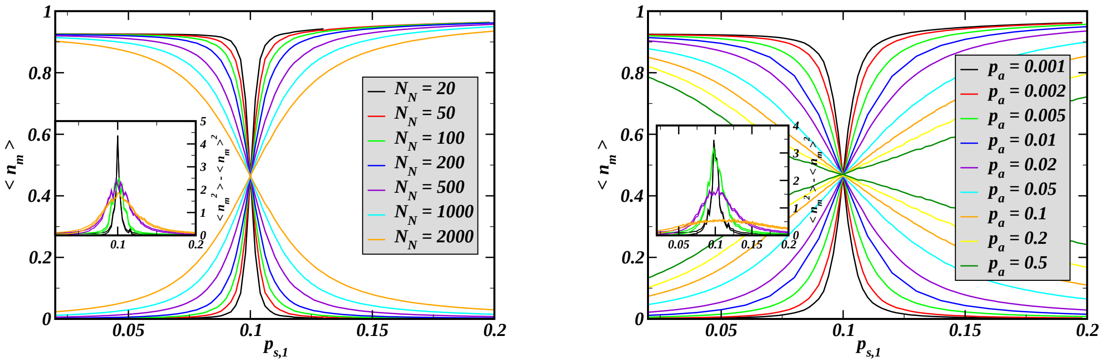

Figure S14. General model behavior. Comparing the average frequency of modifications versus the propagation rate $p_{s,1}$ for different numbers of nucleation sites N_N (left) and for different values of the association rate p_a (right). The inner panels exhibit the temporal fluctuations $< n_m^2 > - < n_m >^2$ in the system for each mark. The system exhibits a behavior similar to a phase transition when changing the propagation constant leading to a drastic increase of the fluctuations at the transition point at $p_{s,1} = p_{s,2}$. At this point, the domains actively compete against each other by changing their size and temporally occupying regions that have been previously occupied by the competing mark. The fluctuations become larger for sharper transitions. The transition becomes smoother for smaller numbers of nucleation sites and/or for larger nucleation rates. The other parameters were $p_{s,2} = 0.1$, $p_d = p_a = 0.01$.

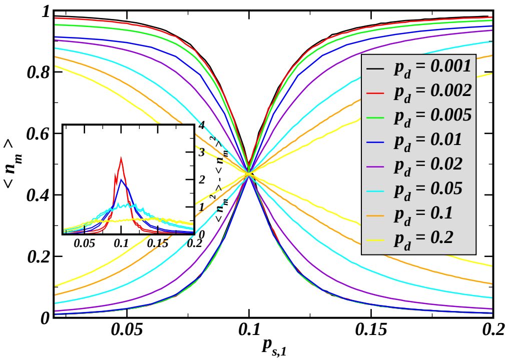

Figure S15. General model behavior. Comparing the average frequency of modifications versus the dissociation rate $p_{s,1}$ for different dissociation rates p_d. The other parameters were $p_s = 0.1, p_a = 0.01, N_N = 1000$.

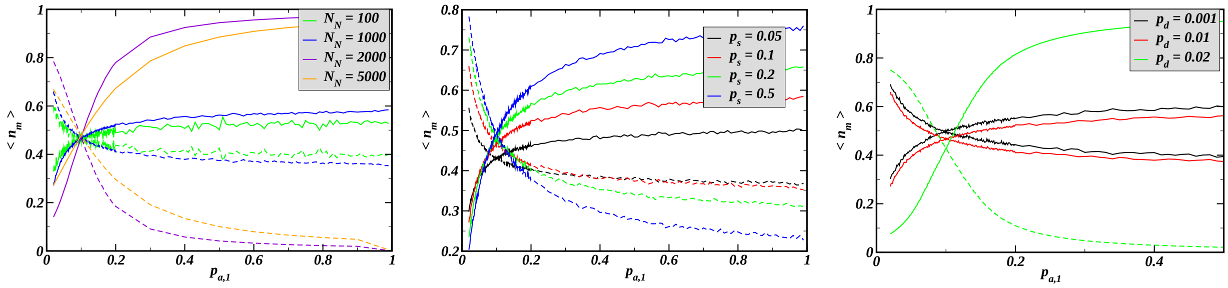

Figure S16. Model behavior for different nucleation rates. We compare the average frequency of modifications versus the association rate $p_{a,1}$ for different numbers of nucleation sites (left), for different values of the propagation rate (center) and for different values of the deletion rate (right). There is no sensitive reaction to a change of the association rate.
